# Racial and Ethnic Disparities in Postpartum Care in the Greater Boston Area During the COVID-19 Pandemic

**DOI:** 10.1001/jamanetworkopen.2022.16355

**Published:** 2022-06-23

**Authors:** Tianyue Mi, Peiyin Hung, Xiaoming Li, Alecia McGregor, Jingui He, Jie Zhou

**Affiliations:** 1 Department of Health Promotion, Education, and Behavior, University of South Carolina, Columbia; 2 Center for Anesthesia Innovation and Quality, Department of Anesthesiology, Perioperative and Pain Medicine, Brigham and Women’s Hospital, Boston, Massachusetts; 3 Department of Health Services Policy and Management, University of South Carolina, Columbia; 4 Department of Health Policy and Management, Harvard T.H. Chan School of Public Health, Boston, Massachusetts; 5 will be with Department of Anesthesiology and Perioperative Medicine, University of Massachusetts School of Medicine, Worcester

## Abstract

**Importance:**

Racial and ethnic disparities in postpartum care access have been well identified in the United States. Such disparities could be exacerbated by the COVID-19 pandemic because of amplified economic distress and compromised social capital among pregnant women who belong to racial or ethnic minority groups.

**Objective:**

To examine whether the COVID-19 pandemic is associated with an increase in the existing racial and ethnic disparity in postpartum care access.

**Design, Setting, and Participants:**

This was a retrospective cohort study using electronic health records data. Multinomial logistic regressions in an interrupted time series approach were used to assess monthly changes in postpartum care access across Asian, Hispanic, non-Hispanic Black (hereafter, Black), non-Hispanic White (hereafter, White) women, and women of other racial groups, controlling for maternal demographic and clinical characteristics. Eligible participants were women who gave live birth at 8 hospitals in the greater Boston, Massachusetts, area from January 1, 2019, to November 30, 2021, allowing for tracking 90-day postpartum access until March 1, 2022.

**Exposures:**

Delivery period: prepandemic (January to December 2019), early pandemic (January to March 2020), and late pandemic (April 2020 to November 2021).

**Main Outcomes and Measures:**

Postpartum care within 90 days after childbirth was categorized into 3 groups: attended, canceled, and nonscheduled.

**Results:**

A total of 45 588 women were included. Participants were racially and ethnically diverse (4735 [10.4%] Asian women, 3399 [7.5%] Black women, 6950 [15.2%] Hispanic women, 28 529 [62.6%] White women, and 1269 [2.8%] women of other race or ethnicity). The majority were between 25 and 34 years of age and married and had a full-term pregnancy, vaginal delivery, and no clinical conditions. In the prepandemic period, the overall postpartum care attendance rate was 75.2%, dropping to 41.7% during the early pandemic period, and rebounding back to 60.9% in the late pandemic period. During the months in the late pandemic, the probability of not scheduling postpartum care among Black (average marginal effect [AME], 1.1; 95% CI, 0.6-1.6) and Hispanic women (AME, 1.3; 95% CI, 0.9-1.6) increased more than among their White counterparts.

**Conclusions and Relevance:**

In this cohort study of postpartum care access before and during the COVID-19 pandemic, racial and ethnic disparities in postpartum care were exacerbated following the onset of the COVID-19 pandemic, when postpartum care access recovered more slowly among Black and Hispanic women than White women. These disparities require swift attention and amelioration to address barriers for these women to obtain much needed postpartum care during this pandemic.

## Key Points

**Question:**

How did postpartum care access change during the COVID-19 pandemic, and were these changes different by maternal race and ethnicity?

**Findings:**

In this cohort study of 45 588 women, the overall postpartum care attendance rate decreased from 75.2% in January to December 2019 (prepandemic) to 41.7% in January to March 2020 (early pandemic) and subsequently rebounded to 60.9% in April 2020 to November 2021 (late pandemic). Black and Hispanic women showed slower reductions in nonscheduling rates during April 2020 to November 2021 compared with their White counterparts.

**Meaning:**

The study highlights racial and ethnic disparities in postpartum care access both before and after the onset of the pandemic, raising concerns about disparities in postpartum care–associated maternal and infant outcomes.

## Teaser

This cohort study examines the association between the COVID-19 and racial and ethnic disparities in postpartum care access.

## Introduction

The postpartum period is a critical time for women to recover from childbirth and adapt to multiple biological, psychological, and social transitions.^1^ This “fourth trimester” is critical for their long-term well-being.^1^ Most importantly, high-quality postpartum care can enhance maternal and infant health^2^ through prevention, early detection, and treatment of physical and mental complications that lead to maternal morbidity and mortality.^3,4^ The American College of Obstetricians and Gynecologists (ACOG) has recommended that women should seek comprehensive postpartum care no later than 90 days after delivery.^5^ However, postpartum care visits may have been interrupted during the COVID-19 pandemic with large-scale social distancing measures.^6,7^ Compared with women who delivered before the pandemic, women who delivered in the early pandemic (April 2020) were 7.8% less likely to attend postpartum care.^8^ Postpartum care interruption might put new mothers at risk of life-threatening health complications, as approximately 61% of maternal deaths occur in the postpartum period.^9^ Women who delay or skip postpartum care miss valuable opportunities to address challenging health concerns,^10^ resulting in frequent emergency department visits, disproportionate hospitalizations,^11^ and undiagnosed postpartum depression.^12^ The COVID-19 pandemic has been evolving and changing dramatically, which can deteriorate pregnancy outcomes post partum.

Prior to the COVID-19 pandemic, racial disparities in postpartum care and maternal health outcomes persisted.^13^ Black mothers had the highest risks and the fastest increasing rate in pregnancy-related mortality and morbidity across all race and ethnicity groups.^14,15^ Compared with White individuals, Black individuals were 3 to 4 times more likely to die from pregnancy-related complications,^16^ had 3-fold higher severe maternal morbidity rates,^17^ and were more than 2 times as likely to be diagnosed with postpartum depression,^18,19,20^ the long-term effects of which would impact their offspring.^21,22^ Lack of adequate postpartum care access likely contributed to these maternal health disparities facing Black patients,^23^ who were 3.5% less likely than White patients to attend postpartum visits.^24^ Nearly half of racial and ethnic minority individuals, compared with only 9% of White individuals, reported unmet postpartum care needs.^25^

During the COVID-19 pandemic, Black and Hispanic pregnant people have been more than twice as likely as non-Hispanic White pregnant people to be infected and/or die from COVID-19,^26^ putting these racial and ethnic minority women at increased risk of adverse postpartum behaviors and outcomes. As stated in the United Nations Secretary-General’s policy, the COVID-19 pandemic has exposed vulnerabilities in social, political, and economic systems, widening preexisting inequalities.^27^ The impacts of the pandemic were amplified among populations that earn less, save less, hold less secure jobs, and have less access to social protection.^27^ The fear of infection risk,^28^ the stress of constrained health care supply,^29^ the limited access to transportation,^7^ and the reduced outside support due to social isolation,^30^—all of which disproportionately affected Black pregnant individuals^31,32^—have complicated postpartum care during the pandemic.

Postpartum care access and its racial disparities during the COVID-19 pandemic have not been well explored,^33^ making it difficult to remedy the disparities in postpartum care access and its associated maternal health burden. Using electronic health records (EHR) data from a large health system, this study aimed to examine the changes in postpartum care access before and during the COVID-19 pandemic, overall and by maternal race.

## Methods

### Data Sources and Study Participants

This study analyzed EHR data of women visiting and giving birth in 8 hospitals with obstetric units in the Mass General Brigham (MGB) system (previously named Partners Health System) in Massachusetts. The EHR provided information on women’s delivery year and month, demographic characteristics, pregnancy-related characteristics, clinical conditions, and maternal residential county, linking to 2017-2019 American Community Surveys^34^ for county-level social vulnerability index.^35^

To obtain access to the data set and ensure confidentiality of patient information, researchers at MGB deidentified patient information and provisionally approved data access by the team at the University of South Carolina (UofSC). The research protocol has been exempted by the institutional review board of both UofSC and MGB. This study followed Strengthening the Reporting of Observational Studies in Epidemiology (STROBE) reporting guideline.

Eligible women (1) gave live birth between January 1, 2019, and November 30, 2021, and (2) had health records from labor and delivery to 90 days post partum. Cases were selected based on delivery month because (1) the time point is the baseline of the outcome of interest (postpartum care access) and (2) the time point reflects the length of COVID-19 exposure during postpartum. Among 50 097 eligible cases, 4509 duplications were removed, yielding a total of 45 588 women in the final analysis.

### Measures

#### Postpartum Care Access

Postpartum care access was identified by the record of postpartum encounter status at 90 days post partum. Encounters were categorized into 3 groups: (1) attended, (2) scheduled but canceled, and (3) never scheduled.

#### Maternal Race and Ethnicity

Race and ethnicity information was recorded in the EHR data. It was categorized into Asian (n = 4735), Hispanic (n = 6950), non-Hispanic Black (hereafter, Black; n = 3399), non-Hispanic White (hereafter, White; n = 28 526), and other races, including American Indian or Alaska Native, Native Hawaiian or other Pacific Islander, and multiple races (n = 1269).

#### Delivery Month

To assess racial and ethnic differences of postpartum care access from January 2019 to November 2021, delivery month was considered continuous with 2 cutoffs: January 2020, when the first US reproductive-age woman was confirmed with COVID-19^26^ and when COVID-19 was declared a public health emergency globally by the World Health Organization (WHO),^36^ and April 2020, when MGB launched units for COVID-19 tests and treatments for patients.^37^ Delivery month was therefore categorized into 3 periods: prepandemic (January to December 2019), early pandemic (January to March 2020), and late pandemic (April 2020 to November 2021).

#### Covariates

Covariates were selected based on Andersen behavioral model of health services use.^38^ Demographic information included age (<18, 18-24, 25-34, 35-44, and ≥45 years) and marital status (single, married or life partner, and other). Pregnancy-related and obstetric characteristics included prenatal care attendance^39^ (timely [if the first prenatal care visit occurred during the first trimester] and delayed [if the first prenatal care visit occurred after the first trimester or no prenatal care] prenatal care), gestational age at delivery (<28, 28-36, and ≥37 weeks), delivery mode (natural vaginal, cesarean, vaginal birth after cesarean [VBAC], and others [eg, induction]). Clinical and behavioral indicators included any severe maternal morbidity,^40^ diabetes, hypertension, depression, and smoking. Residence county–level characteristics included social vulnerability index (bottom quartile, second quartile, third quartile, top quartile). A higher quartile indicates greater negative effects on communities caused by external stresses on human health.^35^

### Statistical Analysis

Descriptive statistics (frequencies and percentages) are presented to describe distributions of postpartum women in terms of their maternal characteristics by race and ethnicity. Bivariate analyses of the association between maternal characteristics and postpartum care access in different time periods were conducted using χ^2^ tests. Multinomial logistic regression in an interrupted time series approach was used to assess monthly changes in postpartum care access across racial groups, controlling for the aforementioned covariates. To compare monthly trends between race and ethnicity subgroups in each of the periods, contrast statements were used for each interaction of race and ethnicity and delivery month.

Descriptive statistics and bivariate analyses were performed with SPSS statistical software version 26.0 (IBM Corp). Multinomial logistic regression was performed with Stata version 15 (StataCorp). Average marginal effects (AMEs) and 95% CIs were estimated. A 2-tailed *P* *<* .05 was used to indicate statistical significance.

## Results

**Figure 1.**
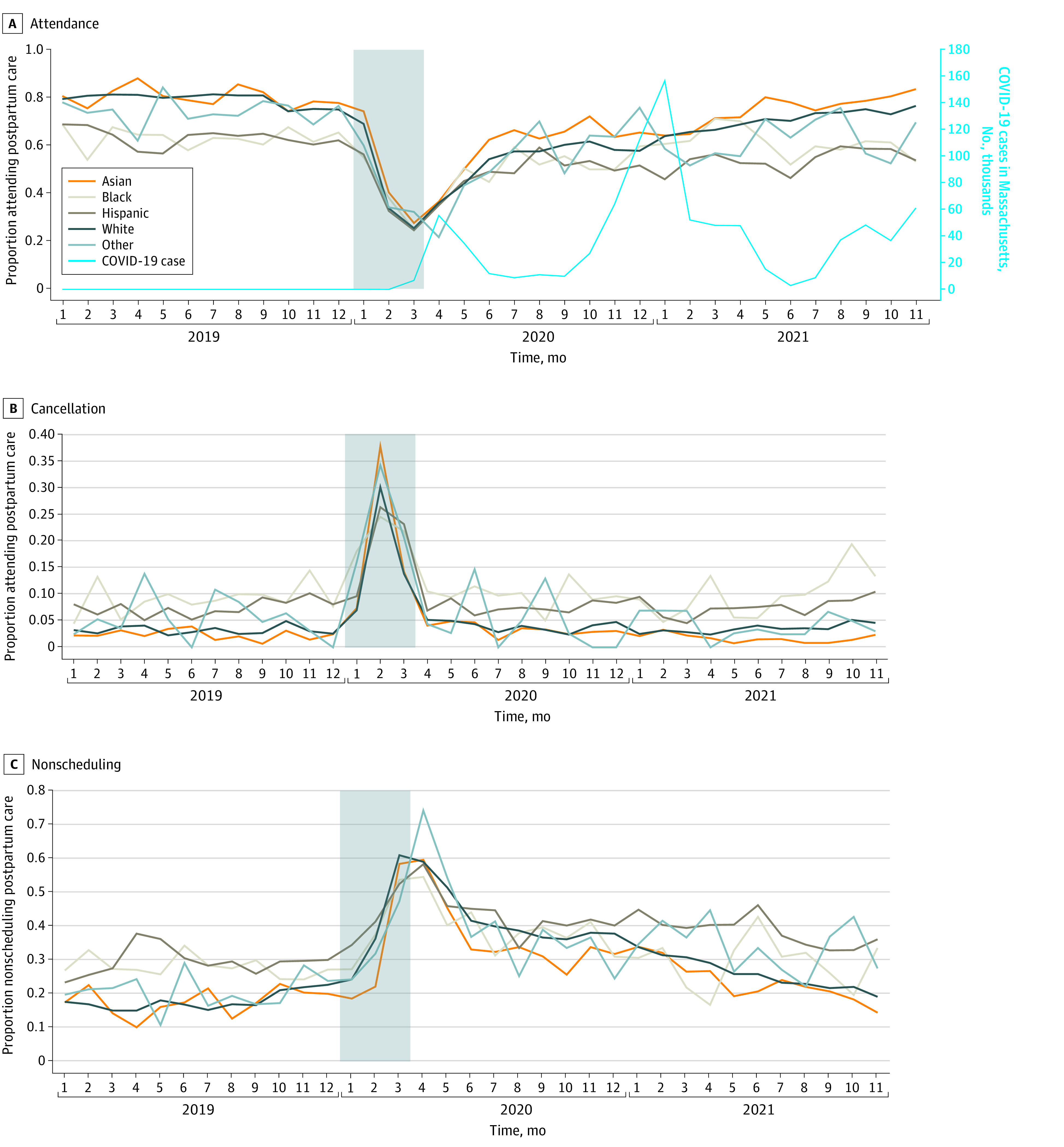
Trends in Postpartum Care Attendance, Cancellation, and Nonscheduling by Delivery Month Between January 2019 and November 2021 Number of COVID-19 cases in Massachusetts per month were derived from Centers for Disease Control and Prevention (CDC).^41^ Shaded area indicates early pandemic period.

The 45 588 women who delivered between January 2019 and November 2021 were racially and ethnically diverse (4735 [10.4%] Asian; 3399 [7.5%] Black; 6950 [15.2%] Hispanic; 28 526 [62.6%] White, and 1269 [2.8%] other races). Overall, women were predominantly aged 25 to 34 years and married and had full-term pregnancies, vaginal deliveries, and no clinical conditions, while the distributions varied across racial and ethnic groups (Table 1). As shown in Figure 1,^41^ the proportion of women attending, canceling, and not scheduling postpartum care within each race and ethnicity group was relatively stable during prepandemic period, from January to December 2019. In the early pandemic (January to March 2020), among all race and ethnicity groups, the attending rate rapidly dropped (from 75.2% to 41.7%), while the canceling and nonscheduling rates correspondingly increased. In the late pandemic (April 2020 to November 2021), the crude attending rate rebounded gradually (to 60.9%), and the crude canceling and nonscheduling rates started to diminish. However, as of November 2021, the crude attending rates were still lower than prepandemic rate within each racial group. Crude cancelation rates as of November 2021 were still higher than those in November 2019.

**Table 1.** Maternal Characteristics by Race and Ethnicity Among 45 588 Women Who Delivered Between January 2019 and November 2021 in the Mass General Brigham System

Characteristic	Race and ethnicity								
	Asian		Black		Hispanic		Other^a^		White, No. (%)
	No. (%)	*P* value^b^	No. (%)	*P* value^b^	No. (%)	*P* value^b^	No. (%)	*P* value^b^	
Childbirths	4735 (10.4)	NA	3399 (7.5)	NA	6950 (15.2)	NA	1269 (2.8)	NA	28 526 (62.6)
Age									
<18	2 (<0.1)	<.001	14 (0.4)	<.001	87 (1.3)	<.001	6 (0.5)	<.001	19 (0.1)
18-24	102 (2.2)		402 (11.8)		1455 (20.9)		140 (11.0)		1028 (3.6)
25-34	2955 (62.4)		1927 (56.7)		3862 (55.6)		720 (56.7)		17 264 (60.5)
35-44	1652 (34.9)		1029 (30.3)		1533 (22.1)		397 (31.3)		10 082 (35.3)
≥45	24 (0.5)		27 (0.8)		13 (0.2)		6 (0.5)		133 (0.5)
Marital status									
Single	357 (7.5)	<.001	1622 (47.7)	<.001	3243 (46.7)	<.001	281 (22.1)	<.001	3913 (13.7)
Married or life partner	4334 (91.5)		1677 (49.3)		3453 (49.7)		945 (74.5)		24 190 (84.8)
Other	44 (0.9)		100 (2.9)		254 (3.7)		43 (3.4)		423 (1.5)
PNC attendance									
Delayed PNC	1325 (28.1)	.25	1567 (46.3)	<.001	2983 (43.1)	<.001	430 (34.0)	<.001	7742 (27.2)
Timely PNC	3398 (71.9)		1817 (53.7)		3942 (56.9)		835 (66.0)		20 676 (72.8)
GA at delivery, wk									
<28	9 (0.2)	.46	30 (0.9)	<.001	28 (0.4)	<.001	0	.04	55 (0.2)
28-36	307 (6.5)		341 (10.1)		597 (8.7)		107 (8.5)		1991 (7.0)
≥37	4387 (93.3)		2989 (89.0)		6271 (90.9)		1154 (91.5)		26 275 (92.8)
Delivery mode									
Natural vaginal	3157 (66.7)	.03	2056 (60.5)	<.001	4642 (66.8)	<.001	811 (63.9)	.03	18 873 (66.2)
Cesarean	1466 (31.0)		1244 (36.6)		2115 (30.4)		432 (34.0)		8978 (31.5)
VBAC	104 (2.2)		95 (2.8)		188 (2.7)		26 (2.0)		550 (1.9)
Other	8 (0.2)		4 (0.1)		4 (0.1)		0		121 (0.4)
Severe maternal morbidity^c^									
Positive	53 (1.1)	.45	88 (2.6)	<.001	101 (1.5)	<.001	15 (1.2)	.52	285 (1.0)
Negative	4682 (98.9)		3311 (97.4)		6849 (98.5)		1254 (98.8)		28 241 (99.0)
Diabetes									
Positive	811 (17.1)	<.001	462 (13.6)	<.001	710 (10.2)	<.001	138 (10.9)	<.001	2142 (7.5)
Negative	3924 (82.9)		2937 (86.4)		6240 (89.8)		1131 (89.1)		26 384 (92.5)
Hypertension									
Positive	302 (6.4)	<.001	525 (15.4)	<.001	709 (10.2)	<.001	129 (10.2)	.02	3551 (12.4)
Negative	4433 (93.6)		2874 (84.6)		6241 (89.8)		1140 (89.8)		24 975 (87.6)
Depression									
Positive	98 (2.1)	<.001	162 (4.8)	<.001	381 (5.5)	<.001	84 (6.6)	.03	2379 (8.3)
Negative	4637 (97.9)		3237 (95.2)		6569 (94.5)		1185 (93.4)		26 147 (91.7)
Smoking									
Positive	8 (0.2)	<.001	40 (1.2)	.55	61 (0.9)	.004	15 (1.2)	.72	371 (1.3)
Negative	4727 (99.8)		3359 (98.8)		6889 (99.1)		1254 (98.8)		28 155 (98.7)
County-level social vulnerability, quartile^d^									
Bottom	35 (0.7)	<.001	40 (1.2)	<.001	146 (2.1)	<.001	20 (1.6)	.001	516 (1.8)
Second	1228 (26.0)		1820 (53.6)		4209 (60.6)		429 (34.0)		8465 (29.7)
Third	256 (5.4)		73 (2.2)		149 (2.1)		38 (3.0)		1313 (4.6)
Top	3205 (67.8)		1461 (43.0)		2442 (35.2)		776 (61.4)		18 183 (63.9)
Hospital									
BWH	1782 (37.6)	<.001	2022 (59.5)	<.001	2944 (42.4)	<.001	493 (38.8)	<.001	9766 (34.2)
CDH	93 (2.0)		37 (1.1)		217 (3.1)		53 (4.2)		1116 (3.9)
MGH	1167 (24.6)		673 (19.8)		1830 (26.3)		343 (27.0)		5687 (19.9)
MVH	5 (0.1)		32 (0.9)		8 (0.1)		68 (5.4)		291 (1.0)
NCH	4 (0.1)		24 (0.7)		95 (1.4)		18 (1.4)		200 (0.7)
NWH	1409 (29.8)		379 (11.2)		518 (7.5)		182 (14.3)		7561 (26.5)
SLM	169 (3.6)		195 (5.7)		1261 (18.1)		66 (5.2)		1375 (4.8)
WDH	105 (2.2)		37 (1.1)		74 (1.1)		46 (3.6)		2518 (8.8)

Abbreviations: BWH, Brigham and Women’s Hospital; CDH, Cooley Dickinson Hospital; GA, gestational age; MGH, Massachusetts General Hospital; MVH, Martha’s Vineyard Hospital; NA, not applicable; NCH, Nantucket Cottage Hospital; NWH, Newton-Wellesley Hospital; PNC, prenatal care; SLM, Salem Hospital; VBAC, vaginal birth after cesarean; WDH, Wentworth Douglass Hospital.

^a^ Other racial groups include American Indian or Alaska Native, Native Hawaiian or other Pacific Islander, and multiple races.

^b^ Differences in maternal characteristics across race groups were calculated with χ^2^ tests, with non-Hispanic White as the reference group.

^c^ Severe maternal morbidity was identified with the US Centers for Disease Control and Prevention list of 21 indicators, including acute myocardial infarction, aneurysm, acute kidney failure, adult respiratory distress syndrome, amniotic fluid embolism, cardiac arrest or ventricular fibrillation, conversion of cardiac rhythm, disseminated intravascular coagulation, eclampsia, heart failure or arrest during surgery or procedure, puerperal cerebrovascular disorders, pulmonary edema or acute heart failure, severe anesthesia complications, sepsis, shock, sickle cell disease with crisis, air and thrombotic embolism, blood products transfusion, hysterectomy, temporary tracheostomy, and ventilation.

^d^ Social vulnerability was indicated by the social vulnerability index and was categorized into quartiles. Higher quartile indicates greater vulnerability.

### Associations Between Maternal Characteristics and Postpartum Care Access Over Time

Black and Hispanic women consistently had lower attending rates over the 3 time periods compared with their White counterparts. From prepandemic to early pandemic periods, the canceling rate among Black and Hispanic women increased from 9.1% to 21.5% and from 7.4% to 19.5%, respectively, while that among White women increased from 3.2% to 16.6%. Yet, from the early pandemic to late pandemic periods, White women had stronger improvements in postpartum care access, with a relatively larger drop in canceling rates (from 16.6% to 3.7%), than Black women (from 21.5% to 10.0%) and Hispanic women (from 19.5% to 7.6%). Most maternal demographic characteristics and clinical conditions were associated with postpartum care access regardless of time periods. For example, women who were married or had a life partner had a higher rate of attending postpartum care than single women. A few maternal characteristics showed varied associations with postpartum care access across time. For example, women who had severe maternal morbidity were significantly less likely to not schedule postpartum care compared with women who had no severe maternal morbidity (28.7% vs 34.5%; *P* < .001) during the late pandemic period, but not during the prepandemic or early pandemic periods (Table 2).

**Table 2.** Bivariate Associations Between Maternal Characteristics and Postpartum Care Access Among 45 588 Women Who Delivered Between January 2019 and November 2021 in the Mass General Brigham System^a^

Characteristic	Postpartum care access											
	Prepandemic period (n = 15 677)				Early pandemic period (n = 3987)				Late pandemic period (n = 25 924)			
	No. (row %)			*P* value	No. (row %)			*P* value	No. (row %)			*P* value
	Attended	Canceled	Nonscheduled		Attended	Canceled	Nonscheduled		Attended	Canceled	Nonscheduled	
Women	11 785 (75.2)	671 (4.3)	3221 (20.5)	NA	1664 (41.7)	717 (18.0)	1606 (40.3)	NA	15787 (60.9)	1214 (4.7)	8923 (34.4)	NA
Race and ethnicity												
Asian	1396 (80.1)	41 (2.4)	305 (17.5)	<.001	201 (47.0)	87 (20.3)	140 (32.7)	.004	1752 (68.3)	64 (2.5)	749 (29.2)	<.001
Black^b^	756 (63.2)	109 (9.1)	331 (27.7)		119 (39.3)	65 (21.5)	119 (39.3)		1071 (56.4)	190 (10.0)	639 (33.6)	
Hispanic	1538 (63.1)	181 (7.4)	717 (29.4)		229 (38.1)	117 (19.5)	255 (42.4)		2034 (52.0)	298 (7.6)	1581 (40.4)	
White	7608 (79.2)	306 (3.2)	1693 (17.6)		1057 (42.4)	413 (16.6)	1024 (41.1)		10 333 (62.9)	612 (3.7)	5480 (33.4)	
Other	340 (74.6)	24 (5.3)	92 (20.2)		39 (40.2)	24 (24.7)	34 (35.1)		419 (58.5)	32 (4.5)	265 (37.0)	
Age, y												
<18	18 (35.3)	9 (17.6)	24 (47.1)	<.001	2 (25.0)	1 (12.5)	5 (62.5)	<.001	23 (28.8)	6 (7.5)	51 (63.8)	<.001
18-24	559 (54.0)	99 (9.6)	378 (36.5)		80 (27.2)	56 (19.0)	158 (53.7)		815 (43.3)	176 (9.3)	892 (47.4)	
25-34	7050 (75.6)	369 (4.0)	1912 (20.5)		1019 (43.2)	388 (16.5)	951 (40.3)		9492 (61.5)	676 (4.4)	5274 (34.2)	
35-44	4084 (78.9)	192 (3.7)	898 (17.4)		555 (42.3)	270 (20.6)	488 (37.2)		5386 (64.0)	352 (4.2)	2674 (31.8)	
≥45	74 (87.1)	2 (2.4)	9 (10.6)		8 (57.1)	2 (14.3)	4 (28.6)		71 (66.4)	4 (3.7)	32 (29.9)	
Marital status												
Single	1883 (61.3)	286 (9.3)	902 (29.4)	<.001	284 (34.5)	164 (19.9)	375 (45.6)	<.001	2849 (50.5)	493 (8.7)	2305 (40.8)	<.001
Married or life partner	9690 (79.3)	360 (2.9)	2167 (17.7)		1343 (43.9)	539 (17.6)	1180 (38.5)		12 699 (64.7)	672 (3.4)	6247 (31.8)	
Other	212 (54.5)	25 (6.4)	152 (39.1)		37 (36.3)	14 (13.7)	51 (50.0)		239 (36.3)	49 (7.4)	371 (56.3)	
PNC attendance												
Delayed	1733 (36.7)	323 (6.8)	2670 (56.5)	<.001	293 (21.4)	142 (10.3)	937 (68.3)	<.001	2419 (29.0)	501 (6.0)	5416 (65.0)	<.001
Timely	9992 (91.9)	345 (3.2)	535 (4.9)		1365 (52.5)	574 (22.1)	663 (25.5)		13 336 (76.1)	711 (4.1)	3467 (19.8)	
GA at delivery, wk												
<28	31 (72.1)	4 (9.3)	8 (18.6)	.005	4 (33.3)	3 (25.0)	5 (41.7)	.07	49 (73.1)	8 (11.9)	10 (14.9)	<.001
28-36	945 (77.5)	86 (7.1)	188 (15.4)		147 (45.2)	71 (21.8)	107 (32.9)		1205 (63.3)	150 (7.9)	548 (28.8)	
≥37	10 741 (75.4)	575 (4.0)	2933 (20.6)		1504 (41.6)	640 (17.7)	1473 (40.7)		14 491 (60.9)	1052 (4.4)	8264 (34.7)	
Delivery mode												
Natural vaginal	7645 (73.7)	427 (4.1)	2300 (22.2)	<.001	1010 (39.3)	467 (18.2)	1090 (42.5)	<.001	10 241 (60.0)	790 (4.6)	6027 (35.3)	<.001
Cesarean	3806 (78.3)	226 (4.6)	830 (17.1)		613 (46.9)	226 (17.3)	467 (35.8)		5205 (62.7)	397 (4.8)	2697 (32.5)	
VBAC	260 (70.8)	18 (4.9)	89 (24.3)		33 (34.4)	22 (22.9)	41 (42.7)		307 (59.4)	27 (5.2)	183 (35.4)	
Other	73 (97.3)	0	2 (2.7)		8 (53.3)	2 (13.3)	5 (33.3)		33 (70.2)	0	14 (29.8)	
Severe maternal morbidity												
Positive	126 (78.3)	8 (5.0)	27 (16.8)	.47	22 (52.4)	8 (19.0)	12 (28.6)	.27	223 (62.8)	30 (8.5)	102 (28.7)	<.001
Negative	11 659 (75.1)	663 (4.3)	3194 (20.6)		1642 (41.6)	709 (18.0)	1594 (40.4)		15 564 (60.9)	1184 (4.6)	8821 (34.5)	
Diabetes												
Positive	1004 (77.2)	76 (5.8)	220 (16.9)	<.001	167 (48.3)	64 (18.5)	115 (33.2)	.01	1735 (64.8)	151 (5.6)	793 (29.6)	<.001
Negative	10 781 (75.0)	595 (4.1)	3001 (20.9)		1497 (41.1)	653 (17.9)	1491 (41.0)		14 052 (60.5)	1063 (4.6)	8130 (35.0)	
Hypertension												
Positive	1261 (78.9)	77 (4.8)	260 (16.3)	<.001	232 (51.2)	66 (14.6)	155 (34.2)	<.001	2162 (66.7)	155 (4.8)	926 (28.6)	<.001
Negative	10 524 (74.7)	594 (4.2)	2961 (21.0)		1432 (40.5)	651 (18.4)	1451 (41.1)		13 625 (60.1)	1059 (4.7)	7997 (35.3)	
Depression												
Positive	715 (73.8)	64 (6.6)	190 (19.6)	.001	93 (40.8)	36 (15.8)	99 (43.4)	.52	1128 (58.0)	149 (7.7)	668 (34.3)	<.001
Negative	11 070 (75.3)	607 (4.1)	3031 (20.6)		1571 (41.8)	681 (18.1)	1507 (40.1)		14 659 (61.1)	1065 (4.4)	8255 (34.4)	
Smoking												
Positive	86 (48.9)	25 (14.2)	65 (36.9)	<.001	17 (28.8)	7 (11.9)	35 (59.3)	.01	92 (34.5)	24 (9.0)	151 (56.6)	<.001
Negative	11 699 (75.5)	646 (4.2)	3156 (20.4)		1647 (41.9)	710 (18.1)	1571 (40.0)		15 695 (61.2)	1190 (4.6)	8772 (34.2)	
Social vulnerability, quartile												
Bottom	205 (78.8)	28 (10.8)	27 (10.4)	<.001	34 (43.6)	18 (23.1)	26 (33.3)	.08	267 (61.2)	44 (10.1)	125 (28.7)	<.001
Second	4126 (71.6)	282 (4.9)	1351 (23.5)		558 (40.5)	258 (18.7)	562 (40.8)		5543 (59.4)	534 (5.7)	3252 (34.9)	
Third	565 (80.1)	33 (4.7)	107 (15.2)		62 (39.2)	40 (25.3)	56 (35.4)		671 (67.6)	73 (7.4)	249 (25.1)	
Top	11 752 (75.2)	666 (4.3)	3219 (20.6)		1661 (41.7)	713 (17.9)	1606 (40.4)		15 762 (60.9)	1210 (4.7)	8921 (34.5)	
Hospital												
BWH	5032 (83.4)	359 (6.0)	639 (10.6)	<.001	775 (53.1)	253 (17.3)	432 (29.6)	<.001	7684 (78.9)	735 (7.6)	1315 (13.5)	<.001
CDH	499 (89.4)	25 (4.5)	34 (6.1)		68 (51.5)	22 (16.7)	42 (31.8)		585 (66.5)	46 (5.2)	249 (28.3)	
MGH	3426 (91.2)	223 (5.9)	106 (2.8)		354 (41.0)	316 (36.6)	194 (22.5)		2814 (53.7)	270 (5.1)	2161 (41.2)	
MVH	1 (0.7)	0	149 (99.3)		0	0	36 (100.0)		0	0	227 (100.0)	
NCH	88 (73.9)	19 (16.0)	12 (10.1)		20 (69.0)	3 (10.3)	6 (20.7)		171 (87.7)	9 (4.6)	15 (7.7)	
NWH	2110 (57.3)	18 (0.5)	1554 (42.2)		302 (34.9)	91 (10.5)	473 (54.6)		2993 (53.9)	80 (1.4)	2477 (44.6)	
SLM	590 (50.6)	26 (2.2)	549 (47.1)		85 (28.8)	32 (10.8)	178 (60.3)		696 (38.9)	73 (4.1)	1018 (57.0)	
WDH	38 (17.7)	0	177 (82.3)		60 (19.9)	0	241 (80.1)		840 (36.6)	1 (<0.1)	1456 (63.4)	

Abbreviations: BWH, Brigham and Women’s Hospital; CDH, Cooley Dickinson Hospital; GA, gestational age; MGH, Massachusetts General Hospital; MVH, Martha’s Vineyard Hospital; NA, not applicable; NCH, Nantucket Cottage Hospital; NWH, Newton-Wellesley Hospital; PNC, prenatal care; SLM, Salem Hospital; VBAC, vaginal birth after cesarean; WDH, Wentworth Douglass Hospital.

^a^ Differences in maternal characteristics across postpartum care access groups were calculated with χ^2^ tests. The prepandemic period was January to December 2019; early pandemic, January to March 2020; and late pandemic, April 2020 to November 2021. Social vulnerability was indicated by the social vulnerability index and was categorized into quartiles. Higher quartile indicates greater vulnerability.

^b^ Other racial groups include American Indian or Alaska Native, Native Hawaiian or other Pacific Islander, and multiple races.

### Level Changes in Early and Late Pandemic Periods Compared With the Prepandemic Period by Race and Ethnicity

As reported in Table 3, compared with the last month in the prepandemic period (December 2019), the probability of canceling and not scheduling postpartum care increased immediately within each race and ethnicity group in the first month of the early pandemic period (January 2020). The probabilities of canceling increased by 8.1 percentage points (95% CI, 4.9-11.2 percentage points; *P* < .001) among Asian women, 5.2 percentage points (95% CI, 0.5-9.9 percentage points; *P* = .03) among Black women, 6.6 percentage points (95% CI, 4.3-8.9 percentage points; *P* < .001) among Hispanic women, and 4.8 percentage points (95% CI, 0.8 to 8.9 percentage points; *P* = .02) among White women; the probabilities of nonscheduling increased by 10.3 percentage points (95% CI, 7.7-13.0 percentage points; *P* < .001) among Asian women, 16.2 percentage points (95% CI, 11.3-21.1 percentage points; *P* < .001) among Black women, 15.0 percentage points (95% CI, 10.1-19.8 percentage points; *P* < .001) among Hispanic women, 16.2 percentage points (95% CI, 13.2-19.1 percentage points; *P* < .001) among White women, and 15.4 percentage points (95% CI, 7.0-23.8 percentage points; *P* < .001) among women of other racial groups. Yet, in the first month of the late pandemic period, the probability of not scheduling postpartum care dropped within each race and ethnicity group compared with the last month in prepandemic period, with a decrease of 2.7 percentage points (95% CI, −3.5 to −2.0 percentage points; *P* < .001) among Asian women, 1.3 percentage points (95% CI, −2.3 to −0.3 percentage points; *P* = .01) among Black women, 1.0 percentage points (95% CI, −1.6 to −0.3 percentage points; *P* = .002) among Hispanic women, 3.0 percentage points (95% CI, −3.3 to −2.8 percentage points; *P* < .001) among White women, and 2.5 percentage points (95% CI, −4.1 to −0.9 percentage points; *P* = .002) among women of other racial groups. Overall, during the late pandemic period, the probability of not scheduling postpartum care among Black women and Hispanic women increased more than among their White counterparts (Black women: AME, 1.1; 95% CI, 0.6-1.6; Hispanic women: AME, 1.3; 95% CI, 0.9-1.6).

**Table 3.** Average Marginal Effects of Cancelling or Nonscheduling Relative to Attending Postpartum Care Visits in Multinomial Logistic Regression^a^

Characteristic	Canceled vs attended		Nonscheduled vs attended	
	Average marginal effect (95% CI)	*P* value	Average marginal effect (95% CI)	*P* value
Race and ethnicity				
Asian	−1.2 (−2.3 to 0.0)	.05	−1.1 (−2.1 to −0.1)	.03
Black	1.9 (−0.4 to 4.2)	.11	1.6 (0.2 to 3.0)	.02
Hispanic	1.9 (−0.2 to 4.0)	.07	3.7 (2.6 to 4.9)	<.001
White	[Reference]		[Reference]	
Other^b^	0.0 (−2.2 to 2.2)	.99	−0.6 (−3.0 to 1.8)	.61
Delivery month	0.1 (0.0 to 0.2)	.06	0.7 (0.5 to 0.8)	<.001
Time period^c^				
Prepandemic	[Reference]		[Reference]	
Early pandemic	1.2 (−1.0 to 3.4)	.27	33.7 (27.8 to 39.5)	<.001
Late pandemic	0.4 (−0.4 to 1.2)	.34	22.2 (20.2 to 24.2)	<.001
**Level changes**				
Early pandemic vs prepandemic				
Asian	8.1 (4.9 to 11.2)	<.001	10.3 (7.7 to 13.0)	<.001
Black	5.2 (0.5 to 9.9)	.03	16.2 (11.3 to 21.1)	<.001
Hispanic	6.6 (4.3 to 8.9)	<.001	15.0 (10.1 to 19.8)	<.001
White	4.8 (0.8 to 8.9)	.02	16.2 (13.2 to 19.1)	<.001
Other^b^	4.5 (−4.2 to 13.2)	.31	15.4 (7.0 to 23.8)	<.001
Late pandemic vs prepandemic				
Asian	−0.1 (−0.4 to 0.1)	.33	−2.7 (−3.5 to −2.0)	<.001
Black	−0.1 (−1.0 to 0.7)	.79	−1.3 (−2.3 to −0.3)	.01
Hispanic	−0.3 (−1.1 to 0.4)	.40	−1.0 (−1.6 to −0.3)	.002
White	0.0 (0.0 to 0.1)	.35	−3.0 (−3.3 to −2.8)	<.001
Other^b^	0.0 (−0.2 to 0.3)	.91	−2.5 (−4.1 to −0.9)	.002
**Changes in monthly trend**				
Prepandemic				
White	[Reference]		[Reference]	
Black	0.1 (−0.2 to 0.5)	.47	−0.3 (−1.0 to 0.4)	.40
Hispanic	0.2 (−0.1 to 0.4)	.24	−0.3 (−0.7 to 0.2)	.22
Asian	−0.1 (−0.2 to 0.1)	.54	0.0 (−0.5 to 0.4)	.84
Other^b^	−0.1 (−0.5 to 0.3)	.75	0.0 (−0.9 to 0.9)	.99
Early-pandemic				
Asian	−1.8 (−7.0 to 3.4)	.49	6.7 (−2.2 to 15.6)	.14
Black	−1.1 (−5.7 to 3.5)	.64	−4.4 (−17.9 to 9.2)	.53
Hispanic	2.7 (−1.0 to 6.3)	.15	−12.0 (−20.5 to −3.5)	.006
White	[Reference]		[Reference]	
Other^b^	−1.0 (−11.5 to 9.5)	.85	−2.3 (−24.5 to 20.0)	.84
Late pandemic				
Black	0.1 (−0.1 to 0.2)	.21	1.1 (0.6 to 1.6)	<.001
Hispanic	0.03 (−0.1 to 0.1)	.56	1.3 (0.9 to 1.6)	<.001
Asian	−0.1 (−0.2 to 0.0)	.17	0.1 (−0.3 to 0.6)	.54
White	[Reference]		[Reference]	
Other^b^	−0.1 (−0.2 to 0.1)	.42	0.4 (−0.2 to 1.0)	.23

^a^ Models controlled for maternal demographic and clinical characteristics, including age, marital status, gestational age at delivery, delivery mode, severe morbidity, diabetes, hypertension, depression, smoking, prenatal care attendance, county-level social vulnerability, and delivery hospital. The full model with covariates can be found in the eTable in the Supplement.

^b^ Other racial groups include American Indian or Alaska Native, Native Hawaiian or other Pacific Islander, and multiple races.

^c^ Prepandemic period was January to December 2019; early pandemic period, January to March 2020; late pandemic period, April 2020 to November 2021.

### Racial and Ethnic Disparity in Monthly Trends of Postpartum Care Access

**Figure 2.**
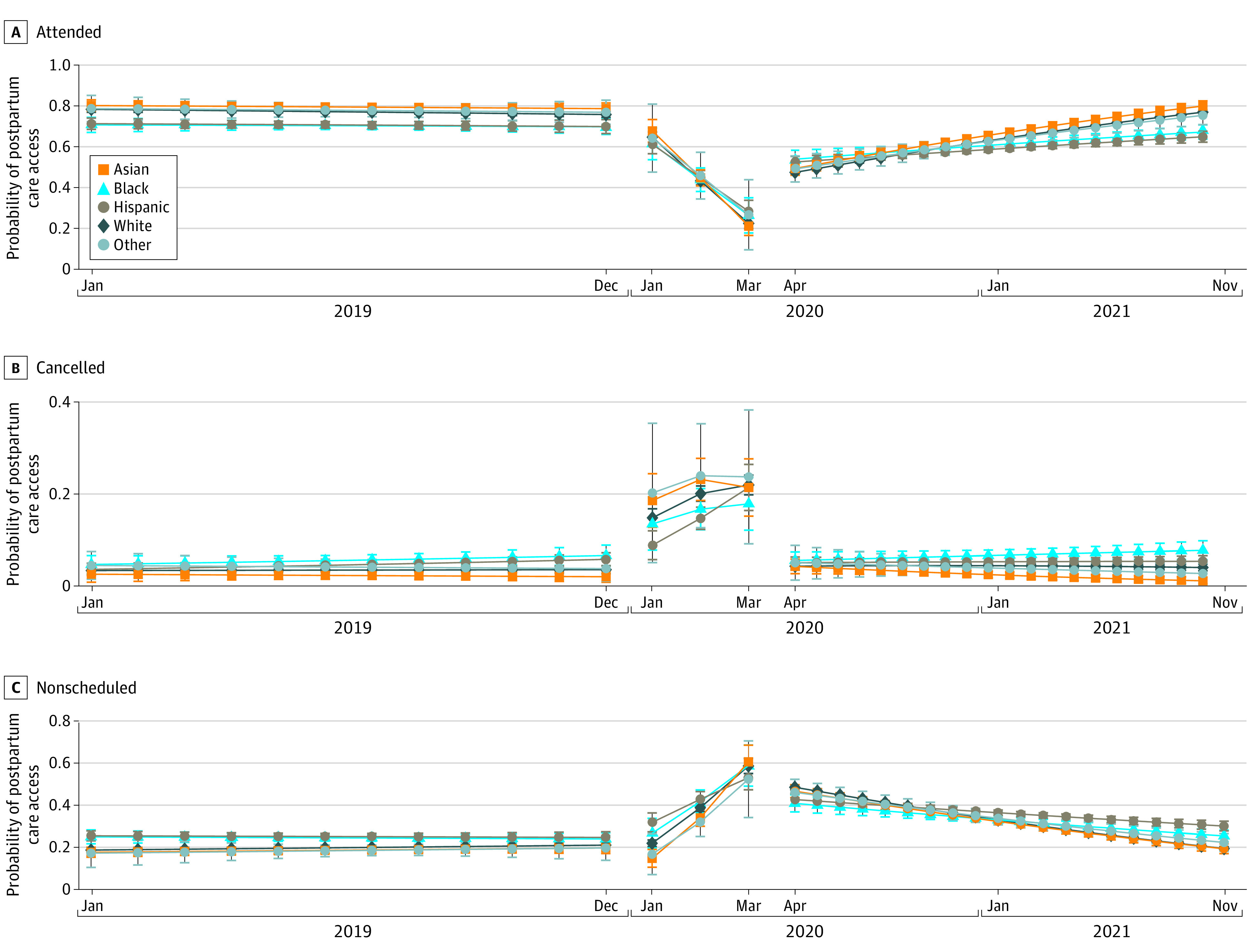
Adjusted Monthly Trends of Postpartum Care Access Probabilities From January 2019 to November 2021 Probabilistic margins of the probabilities of attending, canceling, and nonscheduling postpartum care by delivery month in terms of pandemic phases, estimated using a multinomial logistic model with adjustment for age, marital status, gestational age at delivery, delivery mode, severe morbidity, diabetes, hypertension, depression, smoking, prenatal care attendance, county-level social vulnerability, and delivery hospital, with clustering at the residential zip code level. Whiskers indicate the 95% CIs.

During the prepandemic period (January to December 2019), White women experienced a slightly increasing trend in not scheduling postpartum care; with every 1 month, the probability of not scheduling postpartum care among White women increased by 0.7 percentage points (95% CI, 0.5-0.8 percentage points; *P* < .001) (Figure 2). No differential trend of postpartum care access during the prepandemic and early pandemic periods was found across racial and ethnic groups.

During the late pandemic period, compared with the monthly trends for White women, rates of not scheduling postpartum care among Black women increased more rapidly, with 1.1 percentage (95% CI, 0.6-1.6; *P* < .001) per month. Among Hispanic women, the rate increased 1.3 percentage more rapidly (95% CI, 0.9-1.6; *P* < .001) than White women.

## Discussion

This exploratory study quantified the change in postpartum care access during 3 phases of the COVID-19 pandemic. In the prepandemic period (January to December 2019), the overall postpartum care attendance rate was 75.2%, while it dropped to 41.7% during the early pandemic period (January to March 2020) and rebounded back to 60.9% in the late pandemic period (April 2020 to November 2021). All participating women experienced immediate increases in the probability of postpartum care cancelation or nonscheduling in the early pandemic. Black and Hispanic women experienced a slower reduction in nonscheduling in the late pandemic compared with White women. Higher canceling or nonscheduling rates were also found among women who were younger (18-24 years), were single, had a preterm birth, smoked, and had a delayed prenatal care attendance, compared with older, married, nonsmoking women who initiated prenatal care during the first 12 gestational weeks.

Postpartum care access has been disrupted during the COVID-19 pandemic despite new obstetric precautions being adopted to maintain maternal health care services.^42^ Such disruption is of particular concern given the vital role of access to care on a number of maternal and neonatal health outcomes.^43^ A few studies have documented similar findings to the current study.^8,42^ A pre-post study revealed a 7.8% decrease in postpartum care attendance between April 2019 and April 2020.^8^ A cross-sectional survey study found 62% of pregnant and postpartum women reported health care disruptions due to COVID-19 between May and June 2020.^42^ Of these participants, 29% stated that at least 1 appointment had been canceled^42^ as a result of intentional avoidance of health care facilities,^44^ the feeling of being unsupported and isolated,^45^ concerns around childcare issues, and availability of personal protective equipment.^42^ Extending beyond a few months during the pandemic, the current study found similar postpartum care disruption during the early pandemic but a moderate rebounding trend in postpartum care attendance during the late pandemic.

Although the overall rates of postpartum care attendance, canceling, and nonscheduling have been gradually recovering toward the prepandemic level, adaptation to the COVID-19 pandemic has not been universally equal. The canceling and nonscheduling rates of postpartum care visits decreased more slowly in Black and Hispanic women compared with White women, highlighting the racial and ethnic disparity in adaptation to the pandemic. The delay and absence of postpartum care visits have evidently hindered the prevention against maternal mortality, emergency department visits, the control of childbirth complications, and postpartum depression,^2,11,12^ which were consistently more likely to happen among Black and Hispanic individuals even prior to the pandemic.^16,17,18,19,20^ Little attention has been paid to how intersectional vulnerabilities heighten the risks for serious adverse maternal outcomes, when the populations that suffer long-standing structural inequities are also disproportionately affected by the COVID-19 pandemic.^46^ The current result showed that Black and Hispanic women not only had the highest canceling and nonscheduling rates before the pandemic but also acclimated to the pandemic more slowly than other racial and ethnic groups. These racial and ethnic disparities in postpartum care both before and after the onset of the pandemic raise concerns about the potential increases in disparities in postpartum care–associated maternal and infant outcomes because of the pandemic.

A lack of access to postpartum care was disproportionately prevalent among vulnerable populations, signaling amplified challenges in health care access during the COVID-19 pandemic among the most in need.^12^ This study found that women who were younger than 24 years old, single, multipara, and smoking and who had a preterm delivery were more likely to cancel or not schedule postpartum care visits than their counterparts. In line with previous studies, younger age was associated with postpartum care nonattendance, and it was a particular concern for adolescents.^12,47^ Single or divorced mothers are also well documented to have a higher risk of postpartum care nonattendance compared with married mothers.^48^ Marital status was considered a proxy for spouse support. Encouragement from spouses and/or family members could facilitate women’s ability and motivation to schedule postpartum care visits.^48^ This study highlighted higher cancelation and nonscheduling rates among mothers who smoked and had a preterm birth, in line with previous studies in which low postpartum care attendance was attributed to pregnancy-related factors such as poor birth outcomes.^48,49,50,51^ Efforts aiming to reduce barriers to care and encourage health-seeking behavior are greatly needed to mitigate the negative effects of postpartum care nonattendance among these vulnerable women.

### Limitations

This study has limitations. First, the EHR data were from 8 hospitals in a large health system in Massachusetts. Postpartum care data for women who returned to other care settings were unavailable to us, potentially hindering our ability to differentiate attending postpartum care from nonscheduled postpartum care. Nevertheless, our study revealed different postpartum care access status by maternal race, ethnicity, and SES during the pandemic. Second, insurance information was unavailable. It is well documented that women with Medicaid insurance had a higher risk (37%-55%) of not returning for postpartum care compared with women using private insurance (3%).^47,52^ While higher SES is positively associated with private insurance coverage,^53^ we controlled for county-level income, education, broadband access, and racial residential segregation as the proxies. Despite these limitations, the current study contributes to the extant literature by using longitudinal EHR data in a 24-month timeframe, capturing the trend of postpartum care access before, during, and after the onset of the COVID-19 pandemic. In this racially diverse sample, our results emphasized that racial and ethnic disparities in perinatal care might have been exacerbated by the pandemic. Although overall postpartum care access has gradually rebounded toward the prepandemic level, the attendance rate was still lower than before the pandemic, with Black and Hispanic women lagging behind. Initiatives have been launched to raise awareness and to recommend adaptations during COVID-19 by ACOG,^54^ the Royal College of Obstetrician and Gynecologists,^55^ and WHO.^56^ Yet, some adaptations (eg, temporary birth centers, help hotlines, virtual consultations) might better serve low-risk mothers^57^ than those who require high-acuity care. Our study highlighted the need for greater system-level support from health care professionals and organizations to ensure postpartum care access for vulnerable women and ensure effective adaptation to the pandemic for all.

## Conclusions

This cohort data study highlights exacerbating racial disparities in postpartum care access immediately during and following the onset of the COVID-19 pandemic in early 2020. Of all racial and ethnic groups, Black women showed the slowest reduction in canceling rate, and Hispanic women had the slowest reduction in nonscheduling rate in late pandemic period, indicating slower adaptation to the pandemic. Understanding barriers and facilitators for postpartum care access among Black and Hispanic women is necessary to promote equitable postpartum care access for women most vulnerable to adverse outcomes. Maternal health practitioners and policy makers involved in pandemic adaptation should pay closer attention to avoid potential inequities and the unintentional consequences for marginalized populations.

## Notes

Supplement. **eTable. **Full Model of Multinomial Logistic Regression With Covariates
