# Supplementary material for: Racial and Ethnic Disparities in Postpartum Care in the Greater Boston Area During the COVID-19 Pandemic
Source: JAMA Netw Open. 2022 Jun 23;5(6):e2216355. doi: 10.1001/jamanetworkopen.2022.16355 (PMC9226999; doi:10.1001/jamanetworkopen.2022.16355)
Supplement: Supplement. — eTable. Full Model of Multinomial Logistic Regression With Covariates [file jamanetwopen-e2216355-s001.pdf]

Supplemental Online Content

Mi T, Hung P, Li X, McGregor A, He J, Zhou J. Racial and ethnic disparities in postpartum care in the greater Boston area during the COVID-19 pandemic. *JAMA Netw Open.* 2022;5(6):e2216355. doi:10.1001/jamanetworkopen.2022.16355

eTable. Full Model of Multinomial Logistic Regression With Covariates

This supplemental material has been provided by the authors to give readers additional information about their work.

© 2022 Mi T et al. *JAMA Network Open.*

eTable. Full Model of Multinomial Logistic Regression With Covariates

	Canceled vs. Attended		Non-scheduled vs. Attended	
	Average marginal effects (%) (95% CI)	*P-value*	Average marginal effects (%) (95% CI)	*P-value*
**Race and ethnicity**				
Asian	-1.2 (-2.3, 0.0)	**.05**	-1.1 (-2.1, -0.1)	**.03**
Black	1.9 (-0.4, 4.2)	.11	1.6 (0.2, 3.0)	**.02**
Hispanic	1.9 (-0.2, 4.0)	.07	3.7 (2.6, 4.9)	**<.001**
White	Reference		Reference	
Other[a]	0.0 (-2.2, 2.2)	.99	-0.6 (-3.0, 1.8)	.61
**Delivery month**	0.1 (0.0, 0.2)	.06	0.7 (0.5, 0.8)	**<.001**
**Time period**				
Pre-pandemic	Reference		Reference	
Early-pandemic	1.2 (-1.0, 3.4)	.27	33.7 (27.8, 39.5)	**<.001**
Late-pandemic	0.4 (-0.4, 1.2)	.34	22.2 (20.2, 24.2)	**<.001**
**Level changes**				
Level changes in early-pandemic compared to pre-pandemic by race and ethnicity				
Asian	8.1 (4.9, 11.2)	**<.001**	10.3 (7.7, 13.0)	**<.001**
Black	5.2 (0.5, 9.9)	**.03**	16.2 (11.3, 21.1)	**<.001**
Hispanic	6.6 (4.3, 8.9)	**<.001**	15.0 (10.1, 19.8)	**<.001**
White	4.8 (0.8, 8.9)	**.02**	16.2 (13.2, 19.1)	**<.001**
Other	4.5 (-4.2, 13.2)	.31	15.4 (7.0, 23.8)	**<.001**
Level changes in late-pandemic compared to pre-pandemic by race and ethnicity				
Asian	-0.1 (-0.4, 0.1)	.33	-2.7 (-3.5, -2.0)	**<.001**
Black	-0.1 (-1.0, 0.7)	.79	-1.3 (-2.3, -0.3)	**.01**
Hispanic	-0.3 (-1.1, 0.4)	.40	-1.0 (-1.6, -0.3)	**.002**
White	0.0 (0.0, 0.1)	.35	-3.0 (-3.3, -2.8)	**<.001**
Other	0.0 (-0.2, 0.3)	.91	-2.5 (-4.1, -0.9)	**.002**
**Changes in monthly trend**				
Changes in monthly trends between race and ethnicity groups in pre-pandemic				
Asian	-0.1 (-0.2, 0.1)	.54	0.0 (-0.5, 0.4)	.84
Black	0.1 (-0.2, 0.5)	.47	-0.3 (-1.0, 0.4)	.40
Hispanic	0.2 (-0.1, 0.4)	.24	-0.3 (-0.7, 0.2)	.22
White	Reference		Reference	
Other	-0.1 (-0.5, 0.3)	.75	0.0 (-0.9, 0.9)	.99
Changes in monthly trends between race and ethnicity groups in early-pandemic				
Asian	-1.8 (-7.0, 3.4)	.49	6.7 (-2.2, 15.6)	.14
Black	-1.1 (-5.7, 3.5)	.64	-4.4 (-17.9, 9.2)	.53
Hispanic	2.7 (-1.0, 6.3)	.15	-12.0 (-20.5, -3.5)	**.006**
White	Reference		Reference	
Other	-1.0 (-11.5, 9.5)	.85	-2.3 (-24.5, 20.0)	.84
Changes in monthly trends between race and ethnicity groups in late-pandemic				
Asian	-0.1 (-0.2, 0.0)	.17	0.1 (-0.3, 0.6)	.54
Black	0.1 (-0.1, 0.2)	.21	1.1 (0.6, 1.6)	**<.001**
Hispanic	0.03 (-0.1, 0.1)	.56	1.3 (0.9, 1.6)	**<.001**
White	Reference		Reference	

© 2022 Mi T et al. *JAMA Network Open.*

Other	-0.1 (-0.2, 0.1)	.42	0.4 (-0.2, 1.0)	.23
**Age**				
<18	2.4 (-2.0, 6.7)	.29	1.7 (-4.8, 8.1)	.61
18-24	1.6 (0.1, 3.1)	**.04**	0.3 (-1.1, 1.7)	.65
25-34	Reference		Reference	
35-44	0.1 (-0.3, 0.6)	.57	0.9 (0.0, 1.8)	.06
≥45	-1.6 (-3.6, 0.5)	.14	-0.1 (-5.2, 5.0)	.97
**Marital status**				
Single	2.3 (0.7, 3.9)	**.004**	2.5 (1.6, 3.5)	**<.001**
Married/life partner	Reference		Reference	
Other[a]	2.1 (0.0, 4.1)	**.05**	5.1 (3.0, 7.2)	**<.001**
**PNC attendance**				
Delayed PNC	3.78 (3.42, 4.17)	**<.001**	11.89 (11.19, 12.63)	**<.001**
Timely PNC	Reference		Reference	
**GA at delivery**				
<28	1.7 (-1.7, 5.0)	.34	-4.1 (-11.3, 3.2)	.27
28-36	1.3 (0.1, 2.6)	**.04**	-2.9 (-4.4, -1.4)	**<.001**
≥37	Reference		Reference	
**Delivery mode**				
Natural Vaginal	Reference		Reference	
Cesarean	0.2 (-0.2, 0.6)	.30	-2.9 (-3.7, -2.1)	**<.001**
VBAC	0.6 (-0.7, 1.9)	.37	-0.8 (-4.2, 2.7)	.67
Other	-1.1 (-35.6, 33.5)	.95	-11.4 (-23.5, 0.7)	.07
**Severe Morbidity[b]**				
Positive	0.0 (-1.4, 1.4)	.99	0.1 (-3.4, 3.5)	.97
Negative	Reference		Reference	
**Diabetes**				
Positive	0.9 (0.1, 1.7)	**.03**	-2.7 (-3.9, -1.5)	**<.001**
Negative	Reference		Reference	
**Hypertension**				
Positive	-0.3 (-0.9, 0.3)	.33	-3.5 (-4.5, -2.5)	**<.001**
Negative	Reference		Reference	
**Depression**				
Positive	2.1 (0.3, 4.0)	**.02**	-1.9 (-3.1, -0.8)	**.001**
Negative	Reference		Reference	
**Smoking**				
Positive	3.1 (0.1, 6.0)	**.04**	2.8 (-0.7, 6.2)	.12
Negative	Reference		Reference	
**County-level vulnerability[c]**				
Bottom quartile	Reference		Reference	
Second quartile	-5.3 (-9.3, -1.3)	**.01**	7.2 (4.2, 10.3)	**<.001**
Third quartile	-0.9 (-3.8, 2.1)	.57	-2.2 (-5.4, 1.0)	.17
Top quartile	-4.6 (-8.2, -0.9)	**.01**	3.0 (-0.1, 6.0)	.06
**Hospital**				
BWH	Reference		Reference	
CDH	-4.4 (-5.7, -3.1)	**<.001**	10.1 (7.5, 12.8)	**<.001**
MGH	0.2 (-0.6, 1.0)	.58	15.7 (14.8, 16.7)	**<.001**
MVH	-9.1 (-10.2, -8.0)	**<.001**	84.4 (81.7, 87.1)	**<.001**
NCH	1.5 (-2.1, 5.1)	.41	-2.3 (-6.4, 1.9)	.29
NWH	-7.1 (-8.1, -6.2)	**<.001**	26.7 (25.8, 27.6)	**<.001**
SLM	-4.7 (-5.8, -3.6)	**<.001**	22.7 (21.3, 24.1)	**<.001**
WDH	-9.1 (-10.1, -8.0)	**<.001**	26.5 (24.7, 28.3)	**<.001**

© 2022 Mi T et al. *JAMA Network Open.*

Note. Pre-pandemic: January 2019-December 2019; early-pandemic: January 2020-March 2020; late-pandemic: April 2020-November 2021.

[a]Other racial groups include American Indian or Alaska Native, Native Hawaiian or Other Pacific Islander, and multiple races.

[b]Severe maternal morbidity was identified with the CDC list of 21 indicators, including acute myocardial infarction, aneurysm, acute renal failure, adult respiratory distress syndrome, amniotic fluid embolism, cardiac arrest/ventricular fibrillation, conversion of cardiac rhythm, disseminated intravascular coagulation, eclampsia, heart failure/arrest during surgery or procedure, puerperal cerebrovascular disorders, pulmonary edema/acute heart failure, severe anesthesia complications, sepsis, shock, sickle cell disease with crisis, air and thrombotic embolism, blood products transfusion, hysterectomy, temporary tracheostomy, and ventilation.

[c]Social vulnerability was indicated by the social vulnerability index and was categorized into quartiles. Higher quartile indicates greater vulnerability.

Abbreviations: GA-gestational age; PNC-prenatal care; BWH-Brigham and Women's Hospital; CDH-Cooley Dickinson Hospital; MGH-Massachusetts General Hospital; MVH-Martha's Vineyard Hospital; NCH-Nantucket Cottage Hospital; NWH-Newton-Wellesley Hospital; SLM-Salem Hospital; WDH-Wentworth-Douglass Hospital.

© 2022 Mi T et al. *JAMA Network Open.*
